# Cardiac tamponade presenting as obstructive shock in the first trimester pregnancy – a case report

**DOI:** 10.1186/s12245-026-01120-5

**Published:** 2026-01-20

**Authors:** Dharanidaran Baskaran, K. N. J. Prakash Raju, S. Giridharan, Tharoon Kumar Mathyam Ashok

**Affiliations:** 1 Department of Emergency Medicine, Mahatma Gandhi Medical College and Research Institute, Sri Balaji Vidhyapeeth (Deemed to be University), Puducherry, India; 2 Department of Cardiology, Mahatma Gandhi Medical College and Research Institute, Sri Balaji Vidhyapeeth (Deemed to be University), Puducherry, India; 3 Department of General Medicine, Mahatma Gandhi Medical College and Research Institute, Sri Balaji Vidhyapeeth (Deemed to be University), Puducherry, India

**Keywords:** Obstructive shock, Cardiac tamponade, Pericardial effusion, Maternal mortality, Cardiac arrest

## Abstract

**Background:**

Cardiac tamponade is a critical emergency that needs to be recognized and treated without delay. There is a significant knowledge gap regarding cardiac tamponade in early pregnancy, with no reported cases or guidelines on its evaluation and management. The impact of hemodynamic instability on maternal and fetal outcomes remains unclear.

**Case presentation:**

We present a case of 24-year-old gravida 2 para 1 living 1 (G2P1L1) antenatal mother with no prior co-morbidities who developed sudden cardiovascular collapse secondary to cardiac tamponade caused by massive pericardial effusion. The patient did not survive despite timely aggressive resuscitative efforts, owing to refractory shock from severe myopericarditis probably viral origin.

**Conclusion:**

This case highlights the importance of early point – of – care ultrasound (POCUS) evaluation of pregnant female presenting with unexplained hemodynamic instability and provides critical information on the safety and efficacy of pericardiocentesis in this unique clinical scenario.

**Supplementary Information:**

The online version contains supplementary material available at 10.1186/s12245-026-01120-5.

## Introduction

Cardiac tamponade is a critical emergency that needs to be recognized and treated without delay. While prevalence of clinically insignificant pericardial effusion is common in later part of pregnancy; pericardial effusion leading to cardiac tamponade is a rare event. Current available literatures focuses on benign, asymptomatic pericardial effusion which resolves in the postpartum [1, 2]. However, there is significant knowledge gap in the available literature on incidence of pericardial effusion leading cardiac tamponade in early pregnancy, impact of hemodynamic instability on maternal and fetal outcome as no reported cases were found. And there are no guidelines on evaluation and management of cardiac tamponade in early pregnancy. Our case contributes valuable insight by documenting one of first reported pericardial effusion lead to cardiac tamponade presenting as obstructive shock in first trimester, highlighting diagnostic challenges and need for prompt intervention. It highlights the importance of early POCUS evaluation in pregnant female with unexplained hypotension. We present a 24 years old gravida 2 para 1 living 1 (G2P1L1) female who developed obstructive shock secondary to cardiac tamponade from fulminant myopericarditis likely of viral origin and progressed to recurrent cardiac arrests with unsuccessful resuscitative measures.

## Case presentation

**Fig. 1:**
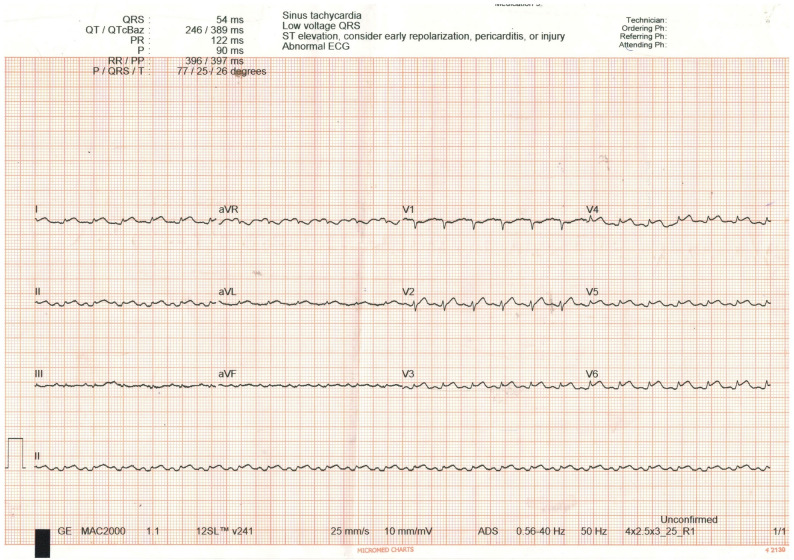
ECG on presentation showing sinus tachycardia, PR segment depression with ST segment elevation in all leads and low voltage QRS in all leads

**Fig. 2:**
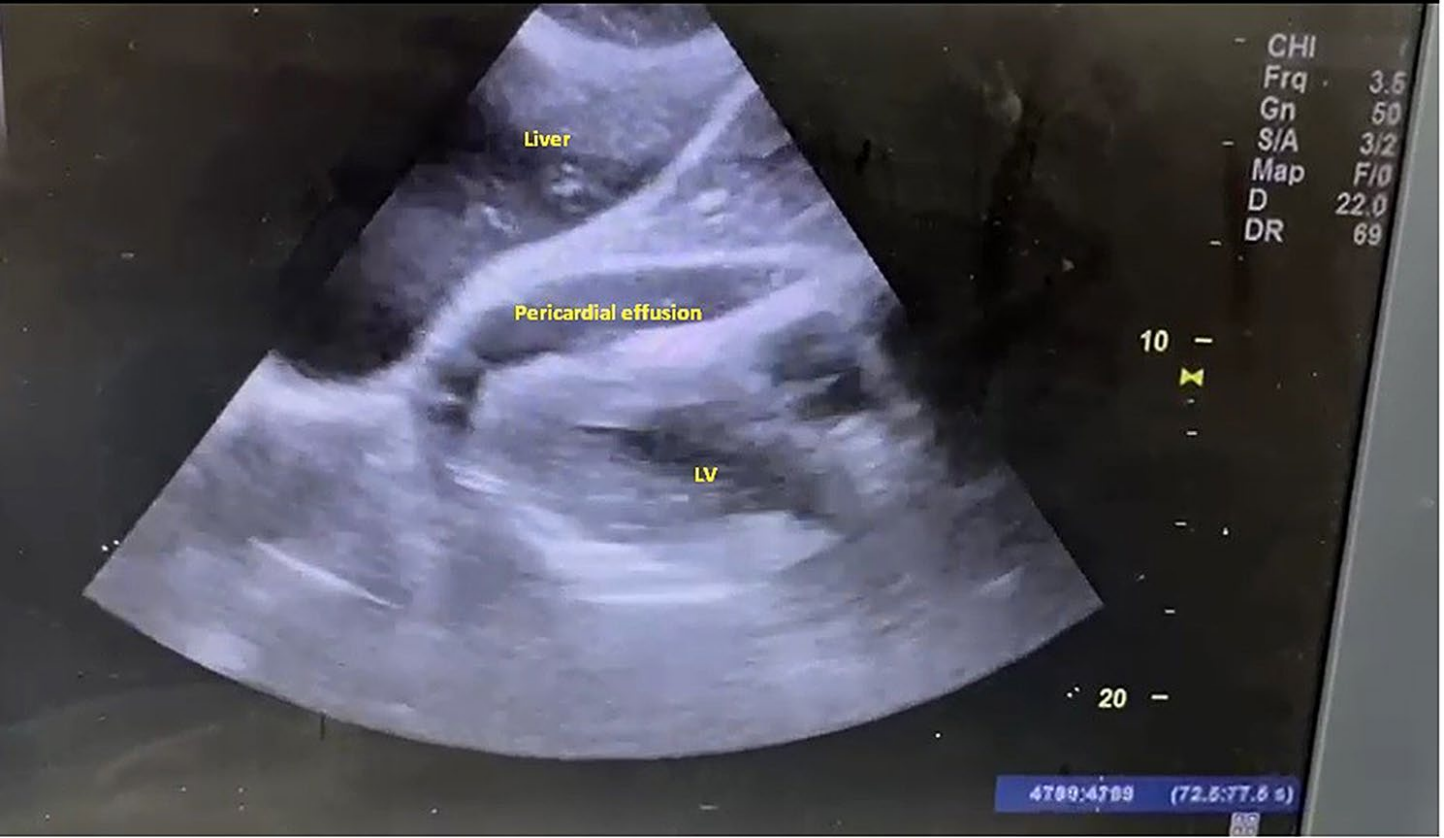
Sub-Xiphoid views of bedside ECHO showing fluid in the pericardial cavity

**Fig. 3:**
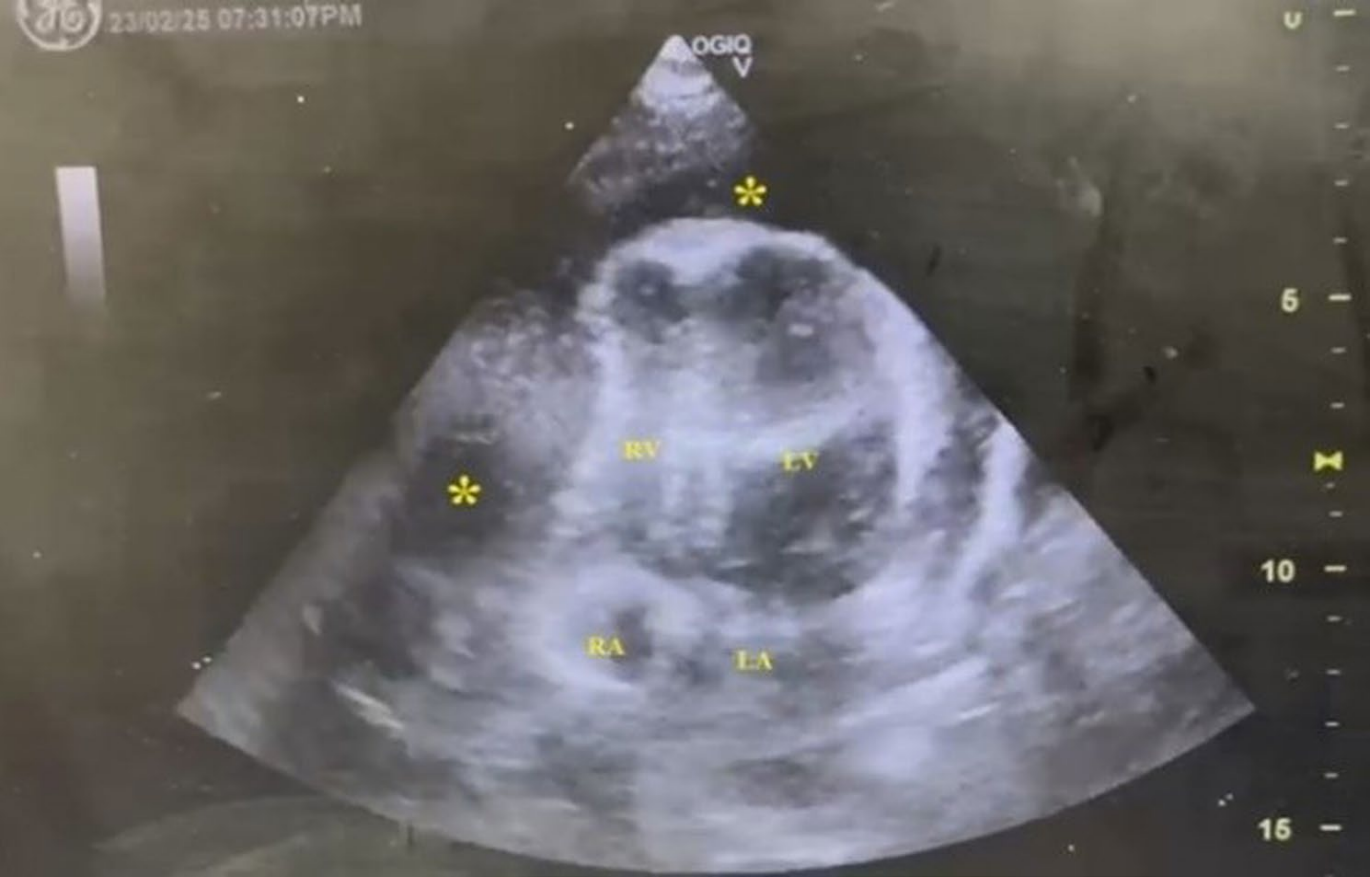
Apical 4 chamber view showing fluid in the pericardial cavity and right ventricular diastolic dysfunction. * - Pericardial effusion

A 24 years old antenatal mother with no prior co-morbid conditions or cardiac disease and uneventful first pregnancy present with New York Heart Association (NYHA) class IV dyspnea and non-specific symptoms like vomiting, giddiness, myalgia from 1 day prior to Emergency Department(ED) visit. On arrival, patient was tachycardic, tachypnoeic and hypotensive. Her limbs were cold and clammy, peripheral pulses were not palpable. She was started on fluid resuscitation. Physical examination of cardiovascular and respiratory system was normal except for diminished first and second heart sounds. ECG (Fig. 1) showed sinus tachycardia with PR segment depression with ST segment elevation and low voltage QRS complex in all leads. POCUS examination (Figs. 2 and 3) of heart revealed massive pericardial effusion (Supplementary Video 1) with right ventricular diastolic collapse with tamponade physiology (Supplementary Video 2). The Inferior Vena cava was dilated with reduced respiratory variation and the Left Ventricular Ejection Fraction was estimated to be 34%. Bedside abdominal ultrasound revealed no ectopic pregnancy, single intrauterine fetus without cardiac activity. Within few minutes of arrival patient went into cardiac arrest, cardiopulmonary resuscitation was started, and endotracheal intubation was done. Immediate ultrasound guided pericardiocentesis done and approximately 100 ml of straw-colored pericardial fluid was drained. After 32 min of Cardio Pulmonary Resuscitation (CPR), Return of Spontaneous Circulation (ROSC) was achieved. Repeat POCUS revealed empty pericardial sac and the estimated Left Ventricular Ejection Fraction after pericardiocentesis was 35% indicating no significant improvement in the systolic function. The patient remained in persistent shock; she was started on multiple vasoactive infusions including Nor-adrenaline, vasopressin and Adrenaline. Despite maximal support she was having refractory hypotension and her blood gases revealed severe mixed respiratory and metabolic acidosis and elevated lactates. Within few hours she again went to cardiac arrest, despite best resuscitative efforts, patient did not survive. Her posthumous reports revealed the following findings: (Tables 1 and 2).

**Table 1:** Blood reports

Parameter	Measured value	Reference Range
**Complete Blood count**		
WBC	9100 cell/cumm	4000–10,000 cell/cumm
Neutrophils	65.9%	40–80%
Lymphocytes	25.1%	20–40%
Hemoglobin	11.7 g/dL	12–15 g/dL
Hematocrit	35.9%	36–46%
Platelets	180,000 cells/cumm	150,000–450,000 cells/cumm
**Basic metabolic Panel**		
Blood Urea	58 mg/dL	12.8–42.8 mg/dL
Creatinine	1.73 mg/dL	0.6–1.2 mg/dL
Sodium	143 mEq/L	138–145 mEq/L
Potassium	4.9 mEq/L	3.5–5.1 mEq/L
Chloride	108 mEq/L	100–107 mEq/L
Calcium	4.3 mg/dL	9–10.2 mg/dL
Magnesium	2.8 mg/dL	1.5–2 mg/dL
Phosphorus	14.5 mg/dL	2.5–4.5 mg/dL
**Liver Function Test**		
Total Protein	2.4 g/dL	6.4–8.2 g/dL
Serum Albumin	1.1 g/dL	3.5–5.2 g/dL
Serum Globulin	1.3 g/dL	2.3–3.6 g/dL
AST	401 U/L	Up to 31 U/L
ALT	563 U/L	Up to 34 U/L
ALP	50 U/L	42–98 U/L
Total Bilirubin	0.2 mg/dL	0.2–1 mg/dL
Direct Bilirubin	0.1 mg/dL	Up to 0.2 mg/dL
Indirect Bilirubin	0.1 mg/dL	0.2–0.6 mg/dL
**Cardiac Enzyme Panel**		
Troponin I	4177.8 ng/L	Up to 19 ng/L
CPK – NAC	1757 U/L	24–170 U/L
CPK – MB	69	1–24 U/L
NT Pro BNP	4118 pg/ml	Up to 125 pg/ml

**Table 2:** Pericardial fluid analysis

Pericardial fluid Analysis	
Total Cell count	acellular
Glucose	148
Triglyceride	98
Adenosine Deaminase activity	9.8
Gram Stain	Occasional pus cells
Culture sensitivity	Sterile
CBNAAT	Negative
ANA	1:100

## Discussion

Our patient is a case of obstructive shock caused by cardiac tamponade during her early gestation leading to recurrent cardiac arrest which caused maternal mortality. Even with multidisciplinary team approach patient did not survive as severe myocardial dysfunction caused persistent and prolonged obstructive shock lead to recurrent cardiac arrest. This case highlights the importance of prompt diagnosis, workup and swift treatment in a pregnant female presenting in unexplained shock.

Cardiac tamponade is rapid accumulation of serous fluid, blood or gas causing compression of cardiac chambers thereby causing impaired diastolic filling, reduced cardiac output and shock which can lead to death [1]. The classical triad of hypotension, raised jugular venous pressure and muffled heart sounds was first described by Beck in 1935 [2]. Other features of cardiac tamponade are tachycardia, pulses paradoxus and low voltages in ECG, increased cardiac silhouette in chest x-ray and demonstration of fluid in pericardial cavity causing abnormal posterior motion of free ventricular wall during diastole.

Cardiac tamponade is rare in pregnancy. However, uncomplicated mild to moderate pericardial effusion can occur in pregnancy which usually resolves without any symptoms by 2 months of post-delivery [3, 4] According to Reddy et al., cardiac tamponade is a progressive phenomenon rather than an ‘all or none’, their study demonstrates that hemodynamic changes can occur early in the disease process and even a small amount of fluid in pericardial cavity can cause tamponade, in our patient 100 mL of pericardial fluid caused tamponade [5]. 

Literature review revealed several case reports on cardiac tamponade due to obstetric and non-obstetric causes during later part of pregnancy and none of the patients in reported cases presented in shock [1, 6–13]. Though Burns E et al. [7], reported a first trimester antenatal mother who had significant pericardial effusion with tamponade physiology, obstructive shock is not the initial presentation and also patient had underlying primary cardiac angiosarcoma. Our case is unique because the patient had no underlying comorbidities and presented during her early pregnancy in obstructive shock. In our patient the cause for cardiac tamponade presenting as obstructive shock is myopericarditis. Pericarditis features were evident on ECG (Diffuse ST segment elevation, PR segment depression). Myocarditis was confirmed by elevated cardiac enzymes, NT-Pro BNP and severe LV dysfunction on Point of Care Ultrasound (POCUS). The probable cause of myopericarditis is viral; as pericardial fluid culture was sterile, Cartridge-Based Nucleic Acid Amplification Test (CBNAAT) was Negative for tuberculosis (Table 1).

Physiological changes occur during pregnancy like increase in blood volume can mask the signs and symptoms of pericardial effusion [14]. Furthermore, symptoms like pedal edema, dyspnea, and hyperventilation can occur in pregnancy as well as in cardiac diseases [1]. Females may be susceptible to pericardial disease during pregnancy because of altered immune response and cardiac dilatation occurring during normal pregnancy which exerts a restraining effect on pericardium. Even subclinical pericardial disease can manifest for the first time during pregnancy [15]. Acute pericarditis is most commonly seen in females of reproductive age. The common causes of pericarditis were summarized in Table 3 [15, 16]. In our case the acute myopericarditis was probably due to viral etiology.

Diagnosing cardiac tamponade is challenging particularly during early pregnancy due to overlap of physiological changes and clinical symptoms and signs. In our case, 24 years old previously healthy pregnant female who experienced sudden onset dyspnoea and arrived in a state of shock, POCUS revealed pericardial effusion with cardiac tamponade. It is noteworthy that pericardium can accommodate larger volume over a period of time, rapid accumulation causes sudden deterioration in hemodynamics with cardiac tamponade with obstructive shock [17]. This rapid accumulation within hours was the probable reason in our patient presenting with obstructive shock.

**Table 3:** ^a^ Common causes of pericarditis

Etiology	Incidence
Idiopathic	85–90%
Infectious Viral	1–2%
Bacterial	1–2%
Tuberculosis	4%
Fungal	Rare
Parasitic	Rare
Neoplasm	7%
Systemic autoimmune disease	3–5%
After cardiothoracic surgery	Rare
Aortic dissection	Rare
Chest wall trauma	Rare
Adverse drug reaction	Rare
Chest wall irradiation	Rare
Acute Myocardial Infarction	—
Myocarditis	—
Uremia Before dialysis	5%
After dialysis started	13%

a – adopted from Elkayam U [15] and Khandekar et al. [16]

Pericardiocentesis to remove the excess fluid from the pericardial cavity is the treatment of choice in cardiac tamponade followed by continuous drainage with indwelling catheter in recurrent pericardial effusions. Pericardiocentesis can be done in the bedside under ultrasound guidance. In our case, despite undergoing urgent bedside pericardiocentesis, the patient’s condition continued to deteriorate. Bedside Echocardiographic assessment showed Left ventricular Ejection fraction of 34% prior to the pericardiocentesis and 35% after the procedure which suggests no significant improvement in the systolic function. Thus the persistent hemodynamic instability and the recurrent cardiac arrest were therefore more likely attributable to prolonged obstructive shock and the metabolic derangements rather than post-procedure ventricular dysfunction.

There is no clinical or echocardiographic evidence of Pericardial Decompression Syndrome such as sudden ventricular dilatation or systolic dysfunction. Postmortem investigations showed sterile pericardial fluid, normal inflammatory markers, and no evidence of bacterial, autoimmune, or malignant etiology. The concurrent fetal demise, following earlier confirmation of a viable pregnancy, was likely due to sustained maternal hemodynamic instability.

Cardiac disease remains a significant contributor to maternal mortality globally. In India, data from the Madras Medical College Pregnancy and Cardiac (M-PAC) registry [18] indicate a maternal cardiac event occurred in 15.2% of pregnancies with heart failure being the most common cardiac event and maternal mortality rate of 1.9% among pregnant women with heart disease, with higher rates observed in those with left ventricular systolic dysfunction (LVSD) and prosthetic heart valves. Although pericardial effusion can occurs during pregnancy which is physiological, benign and self-limiting [1, 2]. In-Hospital mortality of acute pericarditis among general population in developed countries is 1.1% [19], instance like pericarditis complicated by significant effusion and leading to tamponade during pregnancy is rare and data on mortality due to pericardial effusion complicating acute myopericarditis during pregnancy is limited.

## Conclusion

This case highlights the need for a high index of suspicion for cardiac tamponade in a pregnant female with unexplained shock or hemodynamic instability and also emphasizes the importance of POCUS for early diagnosis. Although prompt aggressive fluid resuscitation and pericardiocentesis were performed, persistent hemodynamic instability due to severe myopericarditis led to unfavourable outcome in our patient. Further research needed for standardized protocols for management of pericardial effusion during pregnancy, optimal time of pericardiocentesis and post procedural monitoring for better feto-maternal outcome.

## Limitations

This case report is limited by its description of a single patient, which restricts the generalisability of the findings. Assessment of left ventricular systolic function was based on focused bedside echocardiography rather than a formal comprehensive study. High-quality still images and quantitative echocardiographic measurements could not be obtained because the patient suffered recurrent cardiac arrest and required ongoing emergent resuscitative interventions. A definitive etiological diagnosis could not be established given the acute presentation and the posthumous nature of further investigations. In addition, physiological changes associated with pregnancy may have obscured early clinical signs, potentially contributing to delayed recognition of cardiac tamponade physiology.

## Supplementary Information

Below is the link to the electronic supplementary material.

Supplementary Material 1

Supplementary Material 2

## Data Availability

No datasets were generated or analysed during the current study.

## Abbreviations

ALP: Alkaline Phosphatase
ALT: Alanine Aminotransferase
ANA: Anti–Nuclear Antibody
AST: Aspartate Aminotransferase
CBNAAT: Cartridge–Based Nucleic Acid Amplification Test
CPK–MB: Creatinine Phosphokinase–Myocardial Band
CPK NAC: Creatinine Phosphokinase with N–Acetyl Cysteine
CPR: Cardio–Pulmonary Resuscitation
ED: Emergency Department
ECG: Electrocardiogram
NT Pro BNP: N–terminal–Pro–Brain Natriuretic Peptide
NYHA: New York Heart Association
POCUS: Point Of Care UltraSonography
ROSC: Return Of Spontaneous Circulation
WBC: White Blood Count
